# Evaluating the Impact of Individual and Combined Toxicity of Imidacloprid, Cycloxaprid, and Tebuconazole on *Daphnia magna*

**DOI:** 10.3390/toxics11050428

**Published:** 2023-05-04

**Authors:** Yanli Man, Tian Sun, Chi Wu, Xingang Liu, Mingyuan He

**Affiliations:** 1 State Key Laboratory for Biology of Plant Disease and Insect Pests, Institute of Plant Protection, Chinese Academy of Agricultural Sciences, Beijing 100193, China; 2 Guangxi SPR Technology Co., Ltd., Nanning 530000, China

**Keywords:** ecotoxicity, pesticide mixture, combined toxicity, MIXTOX, risk assessment

## Abstract

The risks posed by chemicals in the environment are typically assessed on a substance-by-substance basis, often neglecting the effects of mixtures. This may lead to an underestimation of the actual risk. In our study, we investigated the effects of three commonly used pesticides—imidacloprid (IMI), cycloxaprid (CYC), and tebuconazole (TBZ)—both individually and in combination, using various biomarkers to assess their impact on daphnia. Our findings indicated that the order of toxicity, from highest to lowest, was TBZ, IMI, and CYC, as determined by acute toxicity as well as reproduction. The effects of the ITmix (IMI and TBZ) and CTmix (CYC and TBZ) combinations on immobilization and reproduction were evaluated by MIXTOX, revealing a higher risk of immobilization at low concentrations for ITmix. The effect on reproduction differed depending on the ration of pesticides in the mixture, with synergism observed, which may be caused mainly by IMI. However, CTmix showed antagonism for acute toxicity, with the effect on reproduction depending upon the composition of the mixture. The response surface also exhibited a switch between antagonism and synergism. Additionally, the pesticides extended the body length and inhibited the development period. The activities of superoxide dismutase (SOD) and catalase (CAT) content was also significantly induced at different dosage points in both the single and combination groups, indicating changes in the metabolic capabilities of detoxifying enzymes and target site sensitivity. These findings highlight the need for more attention to be focused on the effects of pesticide mixtures.

## 1. Introduction

Increasing attention has been paid to the co-existence of pesticides as complex mixtures in the environment due to the lack of comprehensive knowledge and appropriate regulations regarding their combined risks. Neonicotinoids are widely used in modern agricultural practices, and their use is increasing globally [1]. Imidacloprid (IMI), as a representative neonicotinoid, has been widely used since 1991 [2]. However, widespread use of IMI in the field resulted in resistance and cross-resistance in various species [3]. In this case, cycloxaprid (CYC) is used as an alternative to IMI against IMI-resistant pests such as *Aphis gossypii*, *Bemisia tabaci*, and *Nilaparvata lugens* [4,5], and it has broad potential application in the future. Tebuconazole (TBZ) is one of most important fungicides in the world, with its global sales consistently ranking in the top segment of fungicides [6]. Although these pesticides are not applied directly to paddy fields, they may enter water bodies through leaching, runoff, or spray drift after application [4]. IMI has been frequently detected in natural water worldwide, at concentrations of up to 320 μg/L [7,8]. In China, 27% and 84% of water samples collected from the rivers along the east coast exceeded the thresholds of IMI for acute and reproduction-related ecological risks to aquatic organisms, respectively [9]. The concentration of TBZ has been found to reach levels of up to 1.9 μg/L in surface water [10,11]. In addition, in the aquatic environment, IMI and TBZ have been detected as co-occurring mixtures, which may pose a novel synergistic threat to water quality and aquatic organisms simultaneously [12,13]. Concentrations of pollutants in the aquatic environment are more likely to reach levels capable of causing reproduction defects as opposed to acute effects. Previous studies have demonstrated that when mixtures exposed at equipotent concentrations [14,15,16] or in ratios that are not equipotent [17,18] in the aquatic environment, it may cause combined effects, and ignorance of these adverse may result in the risk being underestimated [19,20]. Therefore, public concern relating to environmental chemicals requires extensive risk assessments of various mixtures.

Present risk assessment of the effect of environmental mixtures is chiefly based upon a complex framework of reference models [21]. Normally, combined toxicity is estimated based on the mode of action (MoA) of test chemicals. Mathematical model of concentration addition (CA) is usually used to evaluate the combined toxicity of chemicals with similar toxicity mechanisms. Otherwise, the model of independent action (IA) is used when the chemicals have completely different toxic mechanisms and sites [22]. As environmentally realistic mixtures may be composed of substances that neither act in exactly the same way nor in a strictly dissimilar way, the application of CA and IA estimates to such mixtures has gained substantial attention in the scientific literature. In our study, MIXTOX model was used to analyze the data. This is a stepwise statistical procedure evaluated by either CA or IA, and deviation based on the dose level and chemical ratio dependency from a reference model, which provides significance testing for antagonism, synergism, as well as complex interactions [23], which presents a more reasonable method for the evaluation of combined toxicity in this study.

As a keystone species in the food webs of many continental water bodies, *Daphnia magna* Straus was commonly used in many studies of chemical toxicity in aquatic environments [24]. However, research relating to chemical mixture exposure to daphnia is limited [25]. To date, there have been very few assessment studies conducted involved the mixture of neonicotinoids and TBZ on aquatic organisms under laboratory conditions, especially a mixture of CYC and TBZ. For studies on *Daphnia magna*, only the inhibition of mobilization and reproduction rate tests have been conducted [26,27]. However, the evaluation of survival and/or reproduction may result in the underestimation of the toxicity of some chemicals. Superoxide dismutase (SOD) and catalase (CAT) systems are major antioxidant enzymes defending organisms from oxidative stress. Antioxidant enzyme responses have been reported, including CA and SOD, in response to oxidative tissue damage by UV radiation in daphnia [28,29]. Therefore, parameters including feeding, development time, and oxidative stress, which could impact life history traits should also receive attention [30,31,32].

Establishing a body of knowledge relating to the combined effects of multiple stressors will be helpful for regulatory agencies in the risk assessment of environmental chemicals. Therefore, it is important to assess the risks of mixture on daphnia to indicate response patterns at various levels. We conducted the following experiments: (i) toxicity examination to evaluate the individual and combined toxicity on immobilization and reproduction by MIXTOX method; (ii) determination of body length, development duration, and other reproduction parameters (e.g., first birth time, first spawning number, total brood number) in the reproduction experiment with chronic method; (iii) study of enzyme (SOD and CAT) activities of daphnia expressed in enzyme units (EU) relative to total protein content, upon individual and combined exposure.

## 2. Materials and Methods

### 2.1. Chemicals Used in Testing

IMI (98.5%) and TBZ (97.0%) were purchased from Aladdin Co., Ltd. (Shanghai, China). CYC (96.0%) was provided by Shanghai Shengnong Pesticide Co., Ltd. (Shanghai, China). The catalase assay kits (A007-1 and A024) were obtained from Nanjing Jiancheng Bioengineering Institute. Dimethylformamide (DMF, analytical-grade) was provided by Tianjin Fuyu Fine Chemicals Co., Ltd. (Tianjin, China), and acetonitrile (HLPC grade) was supplied by Thermo Fisher Technology (China) Co., Ltd. (Shanghai, China).

### 2.2. Test Organism Preparation

*Daphnia magna* Straus was obtained from Wuhan Kelduo Biotechnology Co., Ltd. (Wuhan, China) and cultured using Elendt M4 medium in a controlled environmental chamber with a photo-period (16–8 h, light/dark), and a constant water temperature of 20 ± 2 °C. The daphnia were fed daily with *Pseudokirchneriella subcapitata* throughout their culture. In all experiments, neonates (≤24 h-old) were collected as test organisms. The neonates were pooled and randomly divided across the different treatment groups. The sensitivity of daphnia was assessed by potassium dichromate according to OECD 202 guidelines [33], which satisfies the guideline requirement.

### 2.3. Design of Acute and Chronic Exposure Experiments

Acute immobilization tests were conducted following the OECD 202 guideline [33]. The daphnia were exposed to series concentrations of IMI (from 43.00 to 447.00 mg/L), CYC (from 23.00 to 423.00 mg/L), and TBZ (from 3.47 to 13.48 mg/L) with different toxicity ratios. Each treatment was performed in quadruplicate, and five daphnia were used in each replicate. The number of immobilized daphnia was observed at 24 h and 48 h after exposure by gently agitating with test vial and observing the visibility of swimming movements. Daphnia were not fed during the test. At the conclusion of the test, pH, and dissolved oxygen (DO) were measured. Test solutions were collected and measured at 0 h and 48 h of the experiment.

Reproduction toxicity tests were conducted using a semi-static renewal test (renewal period of 2 days) according to the OECD 211 guideline [34]. The concentrations of a single exposure ranged from 1.54 to 10.06 mg/L, 0.96 to 10.06 mg/L, and 0.29 to 0.83 mg/L for IMI, CYC, and TBZ, respectively. Each treatment was conducted across 10 replicates, with one daphnia in each replicate. During the test, daphnia were fed with freshwater algae daily. The number of offspring was observed, then removed daily. The body length of daphnia was recorded at the conclusion of the test. Test solutions were collected and measured at experimental days 0, 2, 4, 7, 14, and 21.

**Figure 1:**
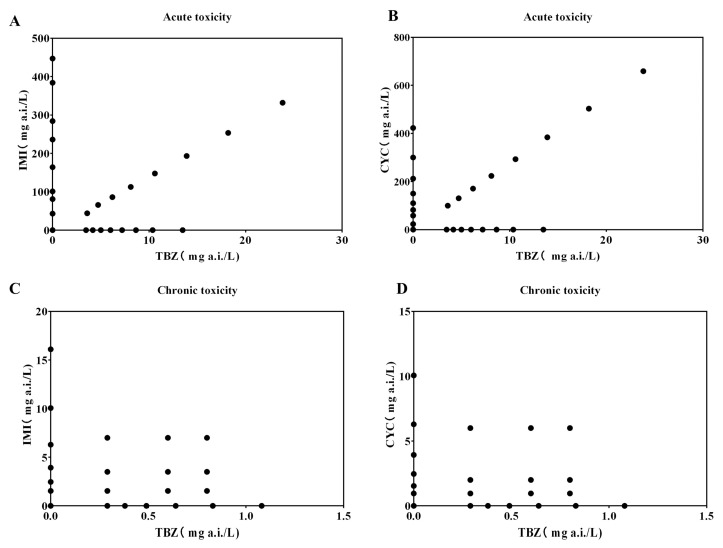
Test design of single and combined acute and reproduction test. (**A**) Acute toxicity under combined exposure of IMI and TBZ; (**B**) Acute toxicity under combined exposure of CYC and TBZ; (**C**) Chronic toxicity under combined exposure of IMI and TBZ; (**D**) Chronic toxicity under combined exposure of CYC and TBZ; TBZ, Tebuconazole; IMI, Imidacloprid; CYC, Cycloxaprid.

For the binary exposure experiment, the tests were conducted using the same procedures in individual exposure tests. The number of replicates used in each treatment was also same as in single chemical exposure tests. The experimental design can be seen in Figure 1. The concentrations in the binary mixtures were selected based on median effective concentration (EC_50_) from the individual exposures. To assess acute toxicity, a fixed-ratio ray was used in the experiment as above. In chronic test, the concentrations for combined toxicity were selected based on the no-observed effect concentration (NOEC) results, as well as the concentrations of approximately 20% and 50% of that used in immobilization testing from individual exposure experiments.

### 2.4. Enzyme Activity Assays

SOD and CAT were selected as candidate enzymes for activity measurements according to kit instructions. At the conclusion of the chronic test, the surviving daphnia were washed twice with distilled water, then wiped and accurately weighed. The *Daphnia magna* were homogenized in a 0.9% sodium chloride solution on ice, then centrifugated at 11,750× *g* for 10 min. Finally, the upper liquor was used to measure enzyme activities using A001-3 and A007-1 assay kits, respectively. All enzyme activities were expressed in enzyme units (EU) relative to total protein content. One EU was defined as the amount of enzyme that degraded one unit of the substrate which was supplied with the kits.

### 2.5. Statistical Analysis

One-way and two-way analysis of variance (ANOVA) and Tukey’s honestly significant difference (HSD) test (*p* < 0.05) were used to analyze statistical significance by SPSS 25.0 (SPSS, Chicago, IL, USA). For assessment of assumptions of normality and homogeneity in parametric analysis, a Levene’s test and Kolmogorov–Smirnov test were used prior to analysis. If the assumptions were not validated, data were log-transformed or ranked. All figures were created using OriginPro 2018 (Northampton, MA, USA).

In the individual exposure experiments, EC_50_, and slope values were calculated using the same dose–response curve formula used in the MIXTOX model [22]. For the data that cannot fit this curve to obtain a reliable EC_50_ estimate, it could be obtained with the Probit Method.

Yi=max1+(CiEC50i)βi

*Y_i_* is the response parameter at a concentration (*C_i_*) of a chemical (*i*); max is the maximum response value; *β* is the slope of the chemical.

For assessment of synergism and antagonism in daphnia exposed to binary mixtures, the MIXTOX tool was used to evaluate possible deviations from the reference models described by Jonker [23], in a Microsoft Excel spreadsheet (http://nomiracle.jrc.ec.europa.eu/Lists/Toolbox/DispForm.aspx%3FID=26.html, accessed on 15 November 2022) provided by the UK Center for Ecology & Hydrology (UKCEH), United Kingdom. These deviations (dose ratio (DR), and dose level (DL)) were computed using two parameters, a and b, forming a nested framework. A detailed description can be found in previously published works [23]. Within each conceptual model, the maximum likelihood method was used for fitting models and potential deviations.

### 2.6. Concentration Verification

Concentrations of IMI, CYC, and TBZ were measured by high performance liquid chromatography (HPLC). Column: Shim-pack GIST C_18_ (100 mm × 2.1 mm, 2 μm); Column oven: 40 °C; Mobile phase: acetonitrile/water (25/75, IMI and CYC; 60/40, TBZ); Flow rate: 0.3000 mL/min; Detector: UV detector SPD-20A; Detection wavelength: 265 nm (IMI), 270 nm (CYC), 225 nm (TBZ); Injection volume: 2 μL; the details of the analytical method validation are provided in the Supplementary Materials, including linear regression and accuracy.

For acute toxicity test, samples from IMI and CYC treatments were diluted 20-fold, and water samples of TBZ were diluted 2-fold with HPLC-grade acetonitrile. The samples were filtered through a 0.22 μm filter membrane prior to HPLC injection. For the reproduction toxicity test, samples from IMI and CYC treatments were diluted 2-fold with HPLC-grade acetonitrile, then filtered through a 0.22 μm filter membrane before HPLC injection. For TBZ, 5 mL of ethyl acetate was added into a centrifuge tube with 5 mL of test sample solution, and then the tube was vortexed. After that, appropriate amount of sodium chloride was added, and then the mixed solution was vortexed again and was allowed to stand and stratify. The upper organic phase was transferred to a spinning bottle. The extraction was repeated once again. The organic phases were mixed for rotary evaporation till nearly dry. An amount of 1 mL of HPLC-grade acetonitrile was added to the spinning bottle, washing the inner wall. The solutions were filtered through a 0.22 μm syringe filter before HPLC injection.

## 3. Results

### 3.1. Concentration in Medium during Exposure

The concentrations of IMI, CYC, and TBZ test solutions throughout the entire experimental workflow in acute and reproduction toxicity tests were in the range of 82–116% of the nominal concentrations of IMI, CYC, and TBZ. The details of the analysis are provided in the Supplementary Materials (Table S1). As there was strong agreement between nominal and actual exposure concentrations, the nominal values of test concentration were used for statistical analysis.

### 3.2. Interaction in Acute Toxicity

#### 3.2.1. Individual Exposure Toxicity

The survival rates of blank and solvent control groups were 90% and 95% at the conclusion of acute bioassay tests, respectively. The EC_50_ and its 95% confidence limits on immobilization of daphnia for the three chemicals are provided in Table 1. The 48 h EC_50_ values of IMI, CYC, and TBZ were 194.0 mg/L, 190.0 mg/L, and 5.74 mg/L, respectively. TBZ had the highest impact on survival compared to others (almost 35-fold higher than the two neonicotinoids, IMI and CYC). However, IMI and CYC had similar effects on daphnia.

#### 3.2.2. Prediction of Combined Toxicity by MIXTOX

Neonicotinoids and TBZ are known to have different MoA, and the IA model would be the optimal model to predict their combined toxicity. Table S2 presents the values for Sum of Squared Residuals (SSR) and *p* (χ^2^), which facilitate the quantification of the model fit and the significance of the deviations from two reference models, respectively.

For the binary ITmix mixture, as shown in Table 2, the IA model was the best fit for indicating additivity. Adding the parameters a and b to the CA model, the SS value significantly increased (*p* (χ^2^) < 0.05). Therefore, the CA model deviated to DL dependence for the best fitting. The proportion of variance from the DL deviation pattern in IA model (99.51%) was slightly higher than that in CA model (99.65%) for this endpoint. Since the slopes were greater than 1, the effect of IA was less severe than that of CA. Therefore, combined with the addition of parameters “a” (a < 0) and “b” (0 < bDL < 1), the toxicity effect on ITmix was synergism at a low dosage level and a switch to antagonism at a high dosage level of above 4 times the EC_50_.

Upon exposure to mixtures of CYC and TBZ, the results of MIXTOX revealed that S/A-dependent deviation from CA model was concluded. Moreover, the parameter a of S/A was positive, which indicated antagonism. For the IA model, by adding parameters a and bDL reduced the SS values significantly (*p* (χ^2^) < 0.05; Table 2), and a DL-dependent deviation from the IA model was therefore noted. Ultimately, combined with the addition parameter of “a” (a > 0) and “b” (bDL < 0), the toxicity effect on CTmix was antagonism depending upon the dosage level. Both CA and IA models indicated that CTmix had antagonistic effects.

### 3.3. Chronic Toxic Effect on Reproduction

#### 3.3.1. Individual Chronic Toxicity

Over the course of the 21-day reproduction exposure, the total number of neonates in the blank control and solvent group was 193 ± 7 and 188 ± 5, respectively. No abnormal behavior was observed in any control groups. As shown in Table 1, the 21d EC_50_ values of IMI, CYC, and TBZ on reproduction are 6.66, 4.56, and 0.70 mg/L, respectively. These results were consistent with acute studies. The effects of CYC and IMI on daphnia have similar effects on reproduction. Additionally, unlike neonicotinoid insecticides, TBZ had higher adverse effects on reproduction. The difference between TBZ and the two neonicotinoids in reproduction was significantly smaller compared to their acute toxicity.

#### 3.3.2. Prediction of Combined Toxicity by MIXTOX

The same procedure was used to analyze the effects of the mixture on reproduction in daphnia. MIXTOX was used to predict the toxicity to reproduction (total number of neonates) of chemical mixtures by deviation of CA and IA (details are shown in Table 3).

**Figure 2:**
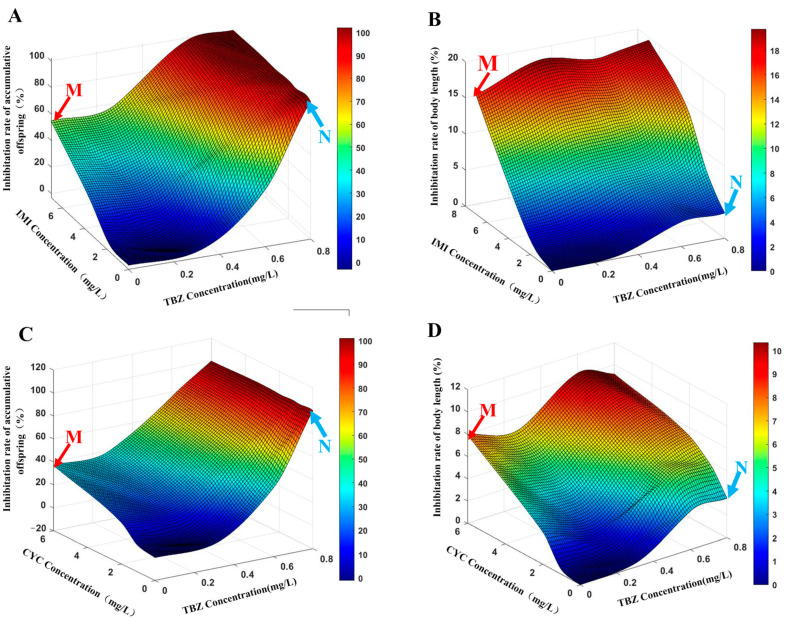
Response surface curve representing inhibition percentage of reproduction (**A**,**C**) and body length (**B**,**D**) of *Daphnia magna* exposed to pesticide mixtures. Points M and N represent the effects of the highest concentrations of tebuconazole and neonicotinoid (imidacloprid and cycloxaprid) tested alone. Color levels represent changes in percentage of inhibition. TBZ, Tebuconazole; IMI, Imidacloprid; CYC, Cycloxaprid.

For the ITmix treatment, DR deviation of IA represented the best fit. Combined with the parameters a (a = 2.18 > 0) and b (b = −13.8 < 0), the effect on reproduction of daphnia was overall antagonism, with synergism observed in the mixture caused mainly by IMI. DR deviation (*p* (χ^2^) < 0.05) of the CA model was fitted after combination with the parameter b. The effect described by the two models was different. However, the slopes in the IA model of DR and the CA model of DR were above 1, and the CA model was more suited to describe the effect on daphnia. In the CA model, a < 0 and b > 0 indicated that the mixture exhibited synergism, and the antagonism observed in the mixture was caused predominantly by TBZ, shown in Figure 2A.

The CTmix group was best described by the reference DR model regardless of the IA or CA models being used. Upon addition of parameters (aDR > 0, bDR < 0), a switch occurred between antagonism and synergism within the response surface depending upon the composition of the mixture. This implies that antagonism can be observed when the toxicity of the mixture is caused mainly by CYC, whereas synergism can be observed where the toxicity is caused mainly by TBZ (bDR < 0), which can be seen in Figure 2C.

#### 3.3.3. Chronic Toxic Interaction Effect on Growth Parameters

**Figure 3:**
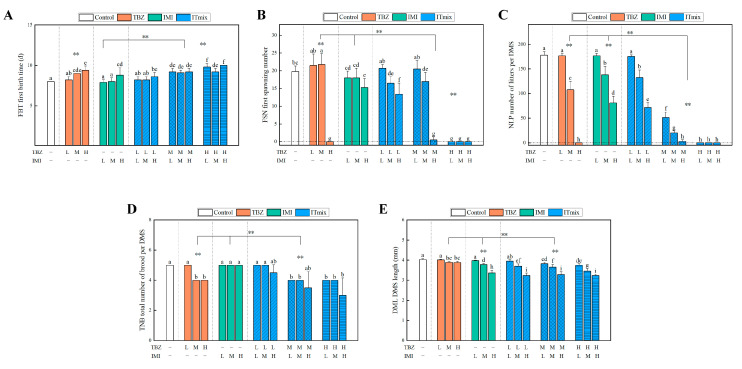
Growth parameters effect by mixture of IMI and TBZ: (**A**) first birth time; (**B**) first spawning number; (**C**) number of litters per daphnia; (**D**) total number of brood per daphnia; (**E**) body length. L: low concentration NOEC; M: medium concentration EC_20_; H: high concentration EC_50_. In the figure, “a–i” indicate significant differences among 15 treatments and control analyzed by one-way ANOVA; “**” indicate the significant differences between the exposure groups and the control, and the results were analyzed by two-way ANOVA (**: *p* < 0.01), The difference in test item exposure (e.g., mixture in difference concentration as a whole group compared with control). TBZ, Tebuconazole; IMI, Imidacloprid; ITmix, mixture of TBZ and IMI.

**Figure 4:**
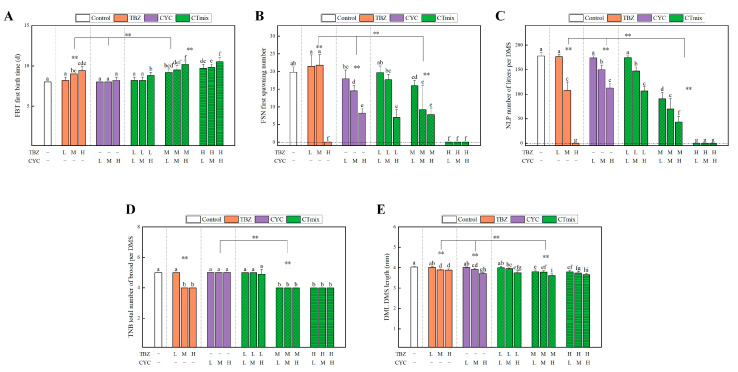
Growth parameters effect by mixture of CYC and TBZ: (**A**) first birth time; (**B**) first spawning number; (**C**) number of litters per daphnia; (**D**) total number of brood per daphnia; (**E**) body length. L: low concentration NOEC; M: medium concentration EC_20_; H: high concentration EC_50_. In the figure, “a–i” indicate significant differences among 15 treatments and control analyzed by one-way ANOVA; “**” indicate the significant differences between the exposure groups and the control, and the results were analyzed by two-way ANOVA (**: *p* < 0.01), the difference in test item exposure (e.g., mixture in difference concentration as a whole group compared with control). TBZ, Tebuconazole; CYC, Cycloxaprid; CTmix, mixture of TBZ and CYC.

**Figure 5:**
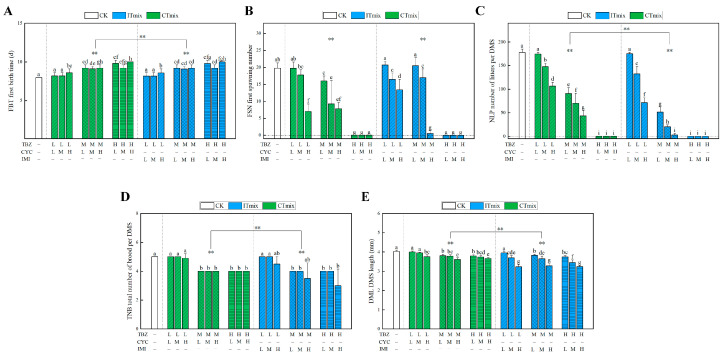
Growth parameters effect by mixture of ITmix and CTmix: (**A**) first birth time; (**B**) first spawning number; (**C**) number of litters per daphnia; (**D**) total number of brood per daphnia; (**E**) body length. L: low concentration NOEC; M: medium concentration EC_20_; H: high concentration EC_50_. In the figure, “a–i” indicate significant differences among 18 treatments and control analyzed by one-way ANOVA; “**” indicate the significant differences between the exposure groups and the control, and the results were analyzed by two-way ANOVA (**: *p* < 0.01), The difference in test item exposure (e.g., mixture in difference concentration as a whole group compared with control). ITmix, mixture of TBZ and IMI; CTmix, mixture of TBZ and CYC.

First birth time, first spawning number, total brood number, and body length of daphnia were observed in the binary mixture groups. The results (Figure 3, Figure 4 and Figure 5) indicated that some parameters were significantly influenced by an increase in concentration. In Figure 3C and Figure 4C, under the concentration recommended by NOEC (0.29 mg/L for TBZ, 1.54 mg/L for IMI, 0.96 mg/L for CYC), the number of neonates per daphnia was slightly decreased; however, this difference was not significant (*p* > 0.05). With increased concentration, the number of neonates per daphnia was significantly decreased (*p* < 0.01), especially when the concentration of TBZ reached 0.80 mg/L (*p* < 0.001), at which point there were no neonates present during the exposure period. After TBZ was combined with CYC and IMI, the total neonate production per daphnia showed a similar change, and the mixture showed a significant change compared with individual exposure and the control group (*p* < 0.01). ITmix had enhanced effects compared to CTmix (*p* < 0.01) (Figure 5C). Poor growth of juveniles was observed when the TBZ concentration reached 0.80 mg/L, which may be a reason for the decline in living newborn daphnia. Some daphnia in the mixture exposure group switched to production of resting eggs through sexual reproduction, which may also relate to a reduction in newborn daphnia.

Additionally, the reduced fecundity may be linked to the brood size and age to maturity of daphnia. Similarly to the effects on total neonates, a reduction in the number of neonates per daphnia was observed at the beginning of the reproductive phase compared to the control. Compared to 20 neonates in the control group, the first spawning number dropped significantly when concentrations of TBZ reached 0.8 mg/L (*p* < 0.01), which are given in Figure 3B and Figure 4B. After treatment with the mixture, there was a significant difference compared to single exposure (*p* < 0.01). Meanwhile, in Figure 5B, the CTmix treatment showed a significant difference when compared to ITmix (*p* > 0.05). Moreover, maturation was negatively affected, leading to a delayed age at first reproduction relating to unexposed daphnids, which is given in Figure 3A and Figure 4A. The time of first birth was slightly prolonged at the NOEC concentration upon single and binary exposure (*p* > 0.05). The time was prolonged alongside an increasing concentration (*p* < 0.05), and it increased to approximately 10 days when TBZ was combined with IMI and CYC. In Figure 5A, there is a significant difference compared to the single exposure and control group (*p* < 0.01), and CTmix had effects on daphnia which were significant different compared to ITmix (*p* < 0.01). Moreover, the total number of broods per daphnia was not significantly altered after treatment with IMI and CYC (*p* > 0.05), regardless of the concentration. The changes in total number of broods in the mixture were similar to other parameters mentioned above (Figure 3D, Figure 4D and Figure 5D).

Similarly to the above findings, there was no significant change in the NOEC group (*p* > 0.05), while the observed change did increase with the concentration (Figure 3E and Figure 4E). Overall, there was a significant difference between ITmix and Ctmix in terms of body length (*p* < 0.01) (Figure 5E). Due to the inhibition of body length being below 50% in all treatment groups, the influence on body length could not be predicted by MIXTOX, and the influence of mixture is presented in Figure 2B,D.

#### 3.3.4. Chronic Toxic Interaction Effect on Enzyme Activities

**Figure 6:**
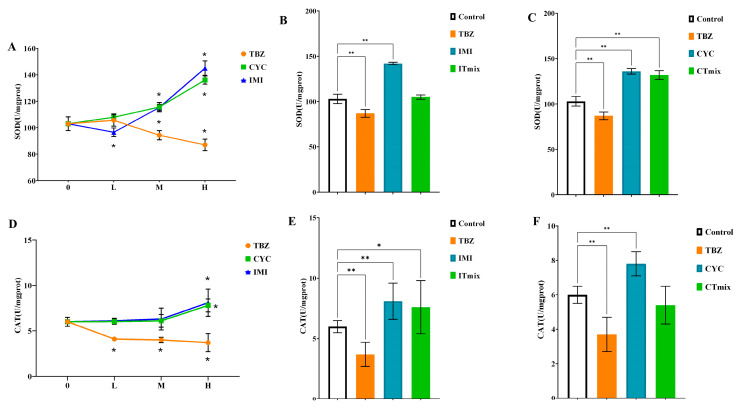
Enzyme content of daphnia exposed to single and combined treatments of IMI, CYC, and TBZ with different treatment (**A**); SOD content treated with single exposure (**B**); SOD content treated with ITmix (**C**); SOD content treated with CTmix (**D**); CAT content treated with single exposure (**E**); CAT content treated with ITmix (**F**); CAT content treated with CTmix. The value of each column represents the mean ± standard deviation (*n* = 10). “* and **” indicate the significant differences between the exposure groups and the control, and the results were analyzed by two-way ANOVA (*: *p* < 0.05; **: *p* < 0.01). ITmix, mixture of TBZ and IMI; CTmix, mixture of TBZ and CYC; L: low concentration NOEC, M: medium concentration EC_20_; H: high concentration EC_50_.

The exposure of daphnia to pesticide treatments caused an alteration in the enzyme activity of SOD and CAT. Considering comparisons to the control treatment, the activity of SOD was induced after exposure with CYC and IMI, and its activity increased with concentration, which shown in Figure 6A,D. Even for the concentration that reached the NOEC of IMI, the SOD enzyme activity was significantly higher than the control group (*p* = 0.045). Similarly to IMI, the SOD enzyme activity was significantly increased after exposure above EC20 of CYC (*p* = 0.033). However, high inhibition of SOD enzyme activity was evident after exposure to TBZ above NOEC, differing from the remaining pesticide treatments (*p* < 0.05). When TBZ was combined with IMI, there was no significant difference compared with the control (*p* > 0.05) (Figure 6F). However, after exposure with CTmix, the stimulation of enzyme activities was significantly higher compared to the control group (*p* < 0.001) (Figure 6C).

The enzyme activity of CAT had similar trends in single exposure groups (Figure 6B,E). The activity of CAT was significantly inhibited after treatment with TBZ (above NOEC). However, the activity was significantly elevated upon isolated exposure to IMI and CYC only under EC_50_ compared with the control group (*p* < 0.001). For combined toxicity of ITmix, the enzyme activity was significantly increased compared with the control (*p* = 0.0472); however, there was no significant difference after TBZ combined with CYC (*p* > 0.05).

## 4. Discussion

Environmental contamination is a growing concern, with many unanswered questions regarding the adverse effects of chemicals on non-target organisms. While some chemicals may individually cause only low, non-significant acute toxic effects at certain concentrations, they can still cause subtle defects in growth, fertility, sex ratios, or reproductive behavior. This phenomenon is particularly pronounced in aquatic life. In this study, the 48 h EC_50_ and 21 d EC_50_ values for single-compound exposures were consistent with previous studies. The neonicotinoids (CYC and IMI) showed similar levels of toxicity on immobilization and reproduction, despite having different activation sites on nicotinic acetylcholine receptors (nAChRs) [35,36]. TBZ had the highest impact on daphnia among three test chemicals, which aligns with previous studies. A study by Qi [37] found that the 48 h EC_50_ values of rac-, R-, and S-TBZ to daphnia were 3.83, 5.34, and 2.35 mg/L, respectively. TBZ may act via a non-receptor-mediated mechanism on non-target organisms, such as anesthesia for acute toxicity, while it inhibits 14-α-demethylase, a fungal P450 cytochrome enzyme involved in the sterol biosynthesis pathway, causing reproductive toxicity [38].

Previous studies have shown the potential for combined effects resulting from exposure to pesticide mixtures, and the neglect of these adverse biological effects may result in underestimation of toxicity [16,17,18]. The toxicokinetics of neonicotinoids IMI and CYC may be similar because they have the same molecular MoA. Although the MoA of TBZ is unclear, it is known to differ from that of neonicotinoids. When beginning with a mixture of IMI and TBZ, the preferred reference model would theoretically be the IA model, because these two chemicals have dissimilar MoAs. However, the toxic effects of co-exposure to neonicotinoid insecticide and TBZ differ in their response patterns between different endpoints (e.g., lethal vs. sublethal). Our results demonstrated that in ITmix, synergism occurs at relatively low concentrations. The result indicated that the joint of IMI and TBZ may be a higher risk at low concentrations. This is inconsistent with current studies on the combined action of multiple compounds, which have demonstrated that synergistic effects occur at relatively high concentrations [39,40]. Synergism at low concentrations has been rarely reported. However, the harm of pesticides is usually caused by long-term accumulation of low doses of pesticides. Low toxicity from low doses of pesticide residues can accumulate through prolonged exposure to organisms that are evidently harmful [41].

The divergence between previous reports on the effects of mixtures is evident in the findings. Some previous studies have indicated that mixtures of neonicotinoid and fungicide have synergistic cumulative effects on pollinators [42,43,44]. The difference in findings may be due to the varied response patterns of mixed pollutants across different species, as mentioned by Gomez-Eyles [45]. Additionally, different ratios of mixed pollutants may have varying influences on organisms. For example, Wang [46] found that different ratios of Cd^2+^ and TiO_2_ had different interaction effects (antagonistic, partially additive, and synergistic) on the green alga Scenedesmus obliquus, similarly to our results. However, the response patterns of pollutant mixtures for different endpoints of reproduction toxicity (offspring per daphnia and body length) were different [47].

In our study, we investigated the long-term exposure effects of a mixture, and we determined the activity of antioxidant enzymes (CAT and SOD). CAT and SOD were induced in a concentration-dependent manner, and the induction rates were significantly higher compared to the control. The results reported in the present study, as well as in previous studies, indicate that antioxidant enzyme responses are transient and variable for different enzymes and chemicals [48]. As mentioned earlier, at the biochemical level, the toxicity of xenobiotics depends on the metabolic capabilities of detoxifying enzymes and target site sensitivity. While it is clear that differences in species exist, there may be some similarities because of evolutionary protection at the biochemical level. One possible explanation for the synergistic effect observed in daphnia is that the co-presence of the two compounds inhibited their respective metabolism, leading to an increase in their toxicity. The decreased synergy at higher doses could be attributed to the induction of additional enzymes.

As we all know, daphnia offspring are sensitive towards xenobiotics, and this sensitivity relies on a variety of factors, including maternal nutrition state, genotype, and clutch size of origin. they are established based on a trade-off mechanism between the quality and quantity of neonates, which are usually controlled by food availability [49]. Therefore, some parameters relating to growth, such as body length and development duration, were influential, and the reason for the observed effects could be related to energy demand [50]. In addition, exposure to sublethal concentrations of Cr (VI) has been reported to induce a reallocation of energy in D. schoderi, allocating less energy to growth but more to detoxification and progeny, which may compensate for toxic stress and ensure the survival of the neonate in an adverse environment [51,52]. The body length was inhibited after exposure, which aligned with previous studies. Overall, the adverse effects on growth, including body length, may be caused by reduced food intake and lower amounts of energy allocated to growth. However, the clear mechanism for this result remains unknown and requires further study.

## 5. Conclusions

In this study, the acute toxicity tests were conducted under lab conditions, and we are surprised to find that the toxicity effect of ITmix was synergism at low dosage levels and was antagonism at high dosage levels of above 4 times the EC_50_. Furthermore, the reproduction tests under sub-lethal concentrations (NOEC, EC_20,_ EC_50_) were performed to evaluate the combined effects on reproduction, developmental duration, and body length. Although the concentrations may be higher than environmentally relevant concentrations, we believe the data can provide thresholds for the field exposure of the two pesticides in practical agriculture and monitoring.

In conclusion, the study demonstrated the negative effects of a mixture of neonicotinoid insecticides with TBZ on *Daphnia magna*. The combined effects of acute toxicity depended on the concentration of the mixture, and it exhibited antagonism which changed to synergism at higher concentrations. In reproduction testing, the effect patterns were dependent on the ratio of the mixture, which exhibited synergism when CYC or IMI were the causative agent of toxicity. These findings showed the complexity of evaluating pesticide toxicity and indicated that traditional toxicological methods such as acute toxicity tests of individuals may result in an underestimation of the environmental impacts of pesticides at low concentrations. When two pesticides with opposite effects were combined, the combined effect on daphnia enzyme activity of daphnia was weaker than the effect of single pesticides. Therefore, more attention should be paid to the assessment of combined toxicity for various research (such as reproduction, developmental, and intergeneration effect). At present, more than 13 products of IMI and TBZ have been registered as combinations in China. The actual concentrations of the two pesticides in the environment are likely to be far beyond the range of the sub-lethal concentrations set in this study, which may have an impact on the ecosystem and human health. The method applied in this study is feasible and can provide theoretical threshold data supporting practical monitoring. Further research on the toxicity effects of binary mixtures of pesticides is important for developing and evaluating predictive mixture models. More studies are needed to reveal the toxic mechanisms from transcriptomic responses.

## Figures and Tables

**Table 1:** The EC_50_ of single exposure of three chemicals to *Daphnia magna*.

Insecticide Type	Exposure Time	Toxic Regression Equation	EC_50_/(mg/L)	95% Confidence Interval	R^2^	NOEC
TBZ	48 h	y = −10.077 + 12.752x	5.74	5.355~6.027	0.9861	
	21 d	y = 1.605 + 10.398x	0.70	0.558~0.935	0.957	0.294
IMI	48 h	y = −26.192 + 11.448x	194.0	183.976~204.008	0.9953	
	21 d	y = −3.628 + 4.406x	6.66	5.999~7.438	0.9843	1.54
CYC	48 h	y = −24.858 + 11.029x	190.0	185.1~195.2	0.9973	
	21 d	y = −2.711 + 4.113x	4.56	3.127~7.588	0.9942	0.96

**Table 2:** Summary of the analysis by MIXTOX of the effects on mobility (48 h) of *Daphnia magna* exposed to the mixtures of TBZ and neonicotinoids (IMI and CYC).

Acute	CA				IA			
	Refer	S/A	DR	DL	Refer	S/A	DR	DL
Vmax	96.18	94.86	102.29	101.71	94.87	95.02	95.10	94.98
*β* _(TBZ)_	3.85	5.50	5.05	2.59	5.33	5.27	5.27	5.24
*β* _(IMI)_	4.28	5.50	3.22	2.55	5.31	5.14	5.13	5.12
EC_50 (TBZ)_	6.96	6.07	14.98	79.18	6.10	6.07	6.07	6.07
EC_50 (IMI)_	226.85	196.32	124.92	118.76	196.90	195.43	195.32	195.49
a	/	2.10	−0.42	−5.15	/	0.29	22.63	0.47
b	/	/	−1.85	0.25	/		−40.81	0.75
SS	5451.51	20,585.91	12,141.54	159,675.57	237.02	230.24	228.90	229.49
χ^2^	/	80.71	81.01	83.50	/	0.75	0.91	0.84
df	/	1.00	2.00	2.00	/	1.00	2.00	2.00
*p* (χ^2^)	/	2.612 × 10^−19^	2.56 × 10^−18^	7.391 × 10^−19^	/	0.39	0.64	0.66
r^2^	0.8434	0.9948	0.9950	0.9965	0.9951	0.9953	0.9953	0.9955
Vmax	104.04	96.07	96.07	95.85	90.59	94.83	94.83	96.93
*β* _(TBZ)_	2.35	4.78	4.78	5.14	4.70	4.92	4.92	4.86
*β* _(CYC)_	0.89	4.41	4.41	4.97	6.95	4.60	4.60	4.52
EC_50 (TBZ)_	6.97	6.05	6.05	6.05	7.41	6.07	6.07	6.02
EC_50 (CYC)_	314.78	183.43	183.43	184.14	233.72	184.49	184.49	182.59
a	/	4.74	4.74	3.81	/	9.70	9.70	1.12
b	/	/	0.00	−0.07	/	/	−1980.93	−7.68
SS	15,806.37	616.74	616.74	518.89	8776.48	693.34	32,033.94	547.01
χ^2^	/	84.59	84.75	89.08	/	65.88	67.19	72.17
df	/	1.00	2.00	2.00	/	1.00	2.00	2.00
*p* (χ^2^)	/	3.67 × 10^−20^	3.95 × 10^−19^	4.53 × 10^−20^	/	4.79 × 10^−16^	2.57 × 10^−15^	2.13 × 10^−16^
r^2^	0.9791	0.9716	0.9702	0.9724	0.7734	0.9705	0.9656	0.9727

/ means that the quantity is not applicable. Vmax, response (growth rate in the absence of both pesticides); β, slope of the individual concentration–response curve for each pesticide; EC_50_ (mg/L), median effective concentration; a and b, parameters in the deviation functions; SS, sum of squared residuals; χ^2^, test statistics; df, degrees of freedom; *p* (χ^2^), the outcome of the likelihood ratio test; CA, concentration addition; IA, independent action; S/A, synergism/antagonism; DR, dose ratio-dependent deviation from the reference; DL, dose level-dependent deviation from the reference.

**Table 3:** Summary of the analysis by MIXTOX for the effects on reproduction of *Daphnia magna* exposed to the mixtures of TBZ and neonicotinoids (IMI and CYC).

Chronic	CA				IA			
	Refer	S/A	DR	DL	Refer	S/A	DR	DL
Vmax	2196.17	2196.45	2198.02	2198.81	206.50	179.97	178.08	186.42
*β* _(TBZ)_	1.68	1.68	1.89	1.61	4.44	5.73	6.79	4.76
*β* _(IMI)_	4.65	4.62	4.72	4.54	1.48	3.16	3.40	2.00
EC_50(TBZ)_	54.35	54.56	50.91	54.17	0.57	0.69	0.74	0.68
EC_50(IMI)_	669.43	671.31	680.18	667.96	5.16	8.00	7.55	7.73
a	/	−0.02	−2.47	0.22	/	−4.57	2.18	−0.03
bcd	/	/	4.96	0.89	/	/	−13.62	−339.9469
SS	383,154.75	383,059.67	258,571.93	381,424.58	12,815.76	7097.33	3804.39	4857.12
χ^2^	/	2.27	17.87	3.77	/	13.00	26.72	21.35
df	/	2.00	1.00	1.00	/	2.00	1.00	1.00
*p* (χ^2^)	/	0.13	1.32 × 10^−4^	0.15	/	3.11 × 10^−4^	1.58 × 10^−6^	2.32 × 10^−5^
r^2^	0.936	0.937	0.996	0.938	0.967	0.912	0.986	0.931
Vmax	171.43	172.17	173.77	172.61	189.03	185.03	173.04	186.63
*β* _(TBZ)_	142.00	2227.12	2197.37	2704.00	6.34	6.57	8.73	6.11
*β* _(CYC)_	1.78	1.58	1.60	1.56	1.45	1.68	3.08	1.46
EC_50 (TBZ)_	0.83	0.83	0.83	0.83	0.64	0.67	0.77	0.67
EC_50 (CYC)_	6.40	6.54	5.51	6.57	4.94	5.50	5.72	5.53
a	/	−0.08	2.35	−0.19	/	−1.27	6.42	−4.11 × 10^−3^
bcd	/	/	−3.14	0.37	/	/	−18.21	−651.76
SS	8436.32	8194.40	5370.80	8138.52	9255.67	8468.10	3130.21	8025.17
χ^2^	/	0.16	23.32	0.18	/	1.96	23.85	3.14
df	/	1.00	2.00	2.00	/	1.00	2.00	2.00
*p* (χ^2^)	/	0.69	8.62 × 10^−6^	0.91	/	0.16	6.62 × 10^−6^	0.21
r^2^	0.863	0.863	0.991	0.865	0.944	0.902	0.995	0.900

/ means that the quantity is not applicable. Vmax response (growth rate in the absence of both pesticides); β, slope of the individual concentration–response curve for each pesticide; EC_50_, (mg/L) median effective concentration; a and b, parameters in the deviation functions; SS, sum of squared residuals; χ^2^, test statistics; df, degrees of freedom; *p* (χ^2^), the outcome of the likelihood ratio test; CA, concentration addition; IA, independent action; S/A, synergism/antagonism; DR, dose ratio-dependent deviation from the reference; DL dose level-dependent deviation from the reference.

## Data Availability

Data is contained within the article or supplementary material.

## Supplementary Materials

The following supporting information can be downloaded at: https://www.mdpi.com/article/10.3390/toxics11050428/s1, Table S1: Nominal and measured concentrations of the additional exercise; Table S2: Interpretation of additional parameters (a and b) form of deviation patterns in MIXTOX.
