# Supplementary material for: Evaluating the Impact of Individual and Combined Toxicity of Imidacloprid, Cycloxaprid, and Tebuconazole on Daphnia magna
Source: Toxics. 2023 May 4;11(5):428. doi: 10.3390/toxics11050428 (PMC10222434; doi:10.3390/toxics11050428)
Supplement: Supplementary file 1 [file toxics-11-00428-s001.zip › toxics-2348383-supplementary.pdf]

Supplementary Material

Evaluation of Individual and Combined Toxicity of Imidacloprid, Cycloxaprid and Tebuconazole on *Daphnia magna*

Yanli Man [1], Tian Sun [2], Chi Wu [1], Xingang Liu [1,*] and Mingyuan He [2,*]

[1] State Key Laboratory for Biology of Plant Disease and Insect Pests, Institute of Plant Protection, Chinese Academy of Agricultural Sciences, Beijing 100193, China
[2] Guangxi SPR Technology Co., Ltd, Nanning 530000, China
* Correspondence: xgliu@ippcaas.cn (X. Liu); hnhmy@126.com (M. He)

1. Quality Control and Method Validation of Analysis Method

Four quality indicators including sensitivity, linearity, reproducibility as well as recoveries were utilized to assess examined to developed analysis method by HPLC (Shimadzu LC-40D XR). The linearity of approach depended on standard solutions in triplicate at series concentrations, 0.1–15.0 mg L^{-1} for IMI, 0.1–20.0 mg L^{-1} for CYC, and 0.8–12.0 mg L^{-1} for TBZ, the correlation coefficients (R^2) were greater than 0.99. Regarding recovery assays, we supplemented proper amount of working normative to blank samples. Spiked degrees were 0.1 mg L^{-1}. Average recoveries of IMI, CYC and TBZ were 92.4–106.5%, 86.0–103.0% and 92.1–103.2%, LOQs were 0.1 mg L^{-1}.

Table S1. Nominal and measured concentrations of the additional exercise.

Table	Nominal Concentration			%	Test Type	Nominal Concentration			%
	TBZ	CYC	IMI			TBZ	IMI	CYC	
Acute	0.00	0.00	0.00	N/A	Chronic	0.000	0	0	N/A
	3.47	0.00	0.00	87–93		0.29	0	0	83–92
	4.17	0.00	0.00	88–97		0.38	0	0	88–99
	5.00	0.00	0.00	85–95		0.49	0	0	83–102
	6.00	0.00	0.00	88–94		0.64	0	0	94–113
	7.20	0.00	0.00	87–93		0.83	0	0	88–110
	8.64	0.00	0.00	89–96		1.08	0	0	84–114
	10.37	0.00	0.00	92–110		0	1.54	0.96	88–112
	13.48	0.00	0.00	94–112		0	2.46	1.54	82–108
	0.00	23.00	43.00	84–104		0	3.93	2.46	92–110
	0.00	58.16	81.00	82–98		0	6.29	3.93	85–114
	0.00	82.00	101.00	83–97		0	10.06	6.29	94–103
	0.00	110.00	164.00	86–92		0	16.10	10.06	99–112
	0.00	150.00	236.00	92–104		0.29	0	0	96–112
	0.00	212.10	284.00	90–112		0.60	0	0	85–104
	0.00	300.00	384.00	89–105		0.80	0	0	80–109
	0.00	423.00	447.00	86–107		0	1.54	0.96	93–110
	3.59	99.60	44.00	86–116		0	3.50	2.00	87–106
	4.72	130.20	65.60	88–111		0	7.00	6.00	88–104
	6.20	170.70	86.00	84–107		0.29	1.54	0.96	89–111
	8.10	223.50	112.60	93–110		0.60	1.54	0.96	92–103
	10.60	293.10	147.60	83–95		0.80	1.54	0.96	88–112
	13.89	383.96	193.20	86–108		0.29	3.50	2.00	86–109
	18.19	502.99	253.30	88–112		0.60	3.50	2.00	92–109

	23.83	658.92	331.82	90–114	0.80	3.50	2.00	88–108
					0.29	7.00	6.00	92–114
					0.60	7.00	6.00	87–104
					0.80	7.00	6.00	86–112

%–Concentration maintain within % of nominal concentration during the test. TBZ–Tebuconazole; IMI–Imidacloprid; CYC–Cycloxaprid.

Table S2. Interpretation of additional parameters (a and b) form of deviation patterns in MIXTOX.

Parameter	CA	IA	Meaning
a	>0	>0	Antagonism
a	<0	<0	Synergism
DR dependence			
a	>0	>0	Antagonism, except for those mixture ratios where significant negative; b indicate synergism
a	<0	<0	Synergism, except for those mixture ratios where significant positive; b indicate antagonism
b	>0	>0	Antagonism where the toxicity of the mixture is caused mainly by toxicant i
b	<0	<0	Synergism where the toxicity of the mixture is caused mainly by toxicant i
DL dependence			
a	>0	>0	Antagonism low dose level and synergism high dose level
a	<0	<0	Synergism low dose level and antagonism high dose level
b	>1	>2	Change at lower dose than the ECs0
b	1	2	Change at the EC_{50} level
b	0<b<1	1<b<2	Change at higher dose level than the EC_{50}
b	<0	<1	No change, but the magnitude of synergism/antagonism is dose level (CA) or effect level (IA) dependent
